# Effects of Sulfate, Chloride, and Bicarbonate on Iron Stability in a PVC-U Drinking Pipe

**DOI:** 10.3390/ijerph14060660

**Published:** 2017-06-19

**Authors:** Jiaying Wang, Tao Tao, Hexiang Yan

**Affiliations:** College of Environmental Science and Engineering, Tongji University, Shanghai 200092, China; wang_123@hotmail.com (J.W.); hxyan@tongji.edu.cn (H.Y.)

**Keywords:** sulfate, chloride, bicarbonate, iron stability, PVC-U drinking pipe, water, models

## Abstract

In order to describe iron stability in plastic pipes and to ensure the drinking water security, the influence factors and rules for iron adsorption and release were studied, dependent on the Unplasticized poly (vinyl chloride) (PVC-U) drinking pipes employed in this research. In this paper, sulfate, chloride, and bicarbonate, as well as synthesized models, were chosen to investigate the iron stability on the inner wall of PVC-U drinking pipes. The existence of the three kinds of anions could significantly affect the process of iron adsorption, and a positive association was found between the level of anion concentration and the adsorption rate. However, the scaling formed on the inner surface of the pipes would be released into the water under certain conditions. The Larson Index (LI), used for a synthetic consideration of anion effects on iron stability, was selected to investigate the iron release under multi-factor conditions. Moreover, a well fitted linear model was established to gain a better understanding of iron release under multi-factor conditions. The simulation results demonstrated that the linear model was better fitted than the LI model for the prediction of iron release.

## 1. Introduction

In drinking water distribution systems (DWDSs), the release of iron from the formed scale in pipes is potentially one of the most important factors affecting the water quality. This process mainly depends on the pH [1], O_2_ solution [2], hardness [3], temperature [4], water velocity [5], soluble solids [6], sediment [7], chlorine [8], cavitation, and erosion [9]. The release and transportation of iron could cause an unpleasant metallic taste, water fouling, and a rusty colour in the DWDSs [10,11,12]. When consulting the drinking standard of China, above 0.3 mg/L for the iron concentration at the consumers’ taps did not measure up.

According to previous studies, anions might be important factors affecting the iron stability in DWDSs, such as sulfate, chloride, and bicarbonate [13,14]. It was reported that a higher concentration of sulfate and chloride generated a greater possibility for the occurrence of red water [15,16]. Bicarbonate, as a buffer, was thought to form a good protective layer on the pipe’s inner surface, which could increase the iron stability in DWDSs [3]. Therefore, in order to describe the comprehensive effects of anions on iron stability in DWDSs, the Larson index (LI) [17], defined as LI = ([Cl^−^] + 2[SO_4_^2−^])/[HCO_3_^−^]), is the most widely used index for considering the anion effects on the metal stability. A higher index of LI indicates a more corrosive body of water. Moreover, different types of drinking pipe material were widely studied for the impact of water quality [18]. In DWDSs, plastic pipes with a small diameter are mainly used; however, metal pipes with a large diameter were predominantly applied in this study. At present, most of the studies on the stability of iron in DWDSs have mainly focused on metal pipes. However, with the extensive use of plastic pipes, the influence of plastic pipes on iron stability should not be ignored.

Plastic pipes, especially those with small diameters (less than 300 mm), are widely used in DWDSs due to their low cost, high structural strength, ease of installation, and corrosion resistance. In China, the drinking water pipes have been replaced in the past few years, comprising 92,300 km of pipes [19], which has meant that a large number of plastic pipes have been applied. Previous researches have also estimated that a large number of Unplasticized poly (vinyl chloride) (PVC-U) resin pipes are used in DWDSs [20].

Our previous studies demonstrated that the inner wall of the PVC-U drinking pipe could adsorb a certain amount of iron from water [21]. Iron release from metal pipes could be one of the sources of iron in DWDSs [11,22]. Meanwhile, the use of iron flocculants during water treatment was also found to be a source of iron [23,24]. As a result, the PVC-U pipes could be considered as a source of secondary iron pollution, thus affecting drinking water quality. Therefore, after long-term use, the interaction between the PVC-U pipe and water could not be ignored and it was of practical significance to study the stability of iron on PVC-U pipes.

In order to produce a better analysis on the influence of PVC-U drinking pipes on the iron stability under different conditions of anions, this paper studied the processes of iron adsorption and release in the following sections. Firstly, the effects of a single factor of anions (sulfate, chloride, and bicarbonate) on iron adsorption and release were analyzed. Secondly, the effect of LI, as the synthetic index of anions, was analyzed on iron release. Thirdly, the trends of iron release by multi-factor analysis were settled. Finally, regression models were obtained to provide qualitative and quantitative support for the impact of PVC-U drinking pipes on iron stability. Therefore, this study will help to promote the current understanding of iron stability on PVC-U drinking pipes under different conditions of anions, thus contributing to the protection of drinking water.

## 2. Materials and Methods

### 2.1. Experimental Set-Up

Several new PVC-U drinking pipes with a diameter of 110 mm were used for the process of iron adsorption, and the prepared PVC-U drinking pipes were used for the iron release in this study. All PVC-U drinking pipes were sealed by horizontal planes with the same pipe material. A solution of 1.5 L with anions (SO_4_^2−^, Cl^−^, HCO_3_^−^) was prepared for use in the study. In the whole experiment, MilliQ water (Millipore Corporation, Bedford, MA, USA) was used. Sodium sulfate (Na_2_SO_4_, ≥99.0%, Sigma-Aldrich, Saint Louis, MO, USA), sodium chloride (NaCl, ≥99.0%, Sigma-Aldrich, Saint Louis, MO, USA), and sodium bicarbonate (NaHCO_3_, ≥99.7%, Sigma-Aldrich, Saint Louis, MO, USA) were used as the sources of SO_4_^2−^, Cl^−^, and HCO_3_^−^, respectively. The pH was maintained at 8.00 (±0.05) by conditioning with hydrochloric acid (HCl, 36.0%–38.0%, Sinopharm Chemical Reagent, Shanghai, China) and sodium hydroxide (NaOH, ≥97.0%, Sigma-Aldrich, Saint Louis, MO, USA). Ferrous sulfate (FeSO_4_·7H_2_O, 99.0%, Sigma-Aldrich, Saint Louis, MO, USA) was used as the source of iron ions during the tests [12].

During the experimental period, 4 mL of aqueous solution was taken by a syphon at 10 cm below the water level each time for iron concentration analysis. All collected samples were acidified with HNO_3_ (3%) and stored in the dark at 4 °C for iron analysis. The iron concentration of the solution was determined by inductively plasma optical emission spectroscopy (ICP-OES) with an Agilent 720es instrument. The water samples in the PVC-U drinking pipes were tested at a rate of 0.1 m/s by using a magnetic stirrer [25].

### 2.2. PVC-U Drinking Pipe Preparation

The PVC-U drinking pipes were prepared for the process of iron release. The procedures for the preparation of the PVC-U drinking pipes were settled as follows. Firstly, the new PVC-U drinking pipes were rinsed with MilliQ water and then air dried for two days (48 h). Secondly, tap water with 8 mg/L iron ion as the test water was added into the PVC-U drinking pipes and stirred at a rate of 0.1 m/s for 10 days. Finally, the prepared PVC-U drinking pipes were ready for the process of iron release after air drying for 12 h.

### 2.3. Iron Adsorption

The tap water and MilliQ water with anions (sulfate, chloride, and bicarbonate) were investigated as the test water during the process of iron adsorption. The initial concentration of iron was 8 mg/L for tap water testing and 0.3 mg/L for MilliQ water using. The test water produced by MilliQ water with sulfate, chloride, and bicarbonate at a concentration of 30, 60, 120, and 250 mg/L was investigated for the single effect of anion condition on iron adsorption.

### 2.4. Iron Release

The same experimental conditions with iron adsorption were used during the process of iron release for an analysis of the effects of a single anion on iron release, except for using the prepared PVC-U drinking pipes (described in Section 2.2) and non-initial iron concentration adding.

To gain a better understanding of iron release, the total trials of 17 synthetic water samples were applied with the prepared PVC-U drinking pipes under multi-factor conditions. According to the standard of drinking water quality, the concentration of sulfate and chloride were researched at the range between 0 and 250 mg/L, and bicarbonate was between 0 and 450 mg/L. The response surface methodology (RSM) was used, which is commonly applied to evaluate individual and interactive effects of independent factors [26,27]. In this research, response surface methodology (RSM) was used to evaluate the trend of iron release on the effects of the three anions (sulfate, chloride, and bicarbonate) under multi-factor water conditions.

### 2.5. Data Analysis

Statistical analyses were carried out using SPSS version 18.0 (IBM Inc., New York, NY, USA). Significant differences between means were determined by Duncan’s multiple range tests and data were subjected to analysis of variance using analysis of variance (ANOVA). The Pearson correlation coefficient was used to estimate linear correlations. The analyses of RSM were used by Design Expert 8 (Stat-Ease Inc., Minneapolis, MN, USA).

## 3. Results

### 3.1. Iron Adsorption under Tap Water Condition

**Figure 1:**
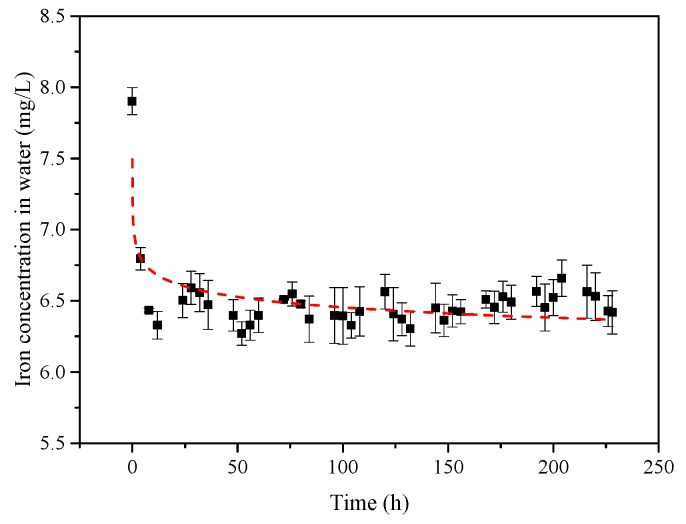
The changes in iron concentration in the test water under stirring condition for 10 days.

With the increase in service life, the inner wall of the PVC-U drinking pipe could adsorb a large amount of iron from the water in the DWDSs. According to our previous study, the scales on the inner wall of PVC-U drinking pipes were mainly found in an oxidized state [21]. Based on the 10 days of iron adsorption, the changes in iron concentration in the water are shown in Figure 1. It can be seen from Figure 1 that the iron concentration dropped rapidly in the first 12 h, and then remained relatively stable.

Therefore, it was possible to calculate the amount of iron adsorption on the inner wall of the PVC-U drinking pipe in this section. The calculated equation is given in Equation (1).

(1) $$M=\frac{(C_{i}-C_{f})\times V}{S}$$

where *M* (mg/cm^2^) represents the amount of iron adsorption on the inner wall of the PVC-U drinking pipe after 10 days of the reaction. *C_i_* (mg/L) and *C_f_* (mg/L) were the initial and final iron concentration in test water, respectively. Meanwhile, *V* (L) represents the volume of test water, and *S* (cm^2^) is the contact area of the pipe and the test water.

After the process of iron adsorption, it could be calculated that a large amount of iron (3.95 × 10^−3^ mg/cm^2^) was attached to the inner wall of the PVC-U drinking pipe, which showed a certain thickness uniformity. The results illustrate that the inner wall of PVC-U drinking pipe could adsorb a large amount of iron from the test water. Moreover, the renewal of the water would aggravate the iron adsorption in the actual pipe network.

In this case, the process of iron adsorption could accumulate iron on the inner wall of the PVC-U drinking pipe, and the pipe could be seen as a source of secondary iron pollution, thus affecting the drinking water quality. Therefore, the study on the iron stability on the PVC-U drinking pipe was of great significance to ensure the drinking water quality.

### 3.2. Iron Adsorption under the Single Factor of the Three Anions

In this part, depending on the new PVC-U drinking pipes, sulfate, chloride, and bicarbonate were studied for the single effect of an anion on iron adsorption after 12 h of the reaction. The iron adsorption rate could be calculated by Equation (2).

(2) $$R_{measured}=\frac{C_{i}-C_{f}}{C_{i}}\times 100\%$$

where *R_measured_* (%) represents the iron adsorption rate after 12 hours of the reaction, *C_i_* (mg/L) is the initial iron concentration, and *C_f_* (mg/L) is the final iron concentration.

**Figure 2:**
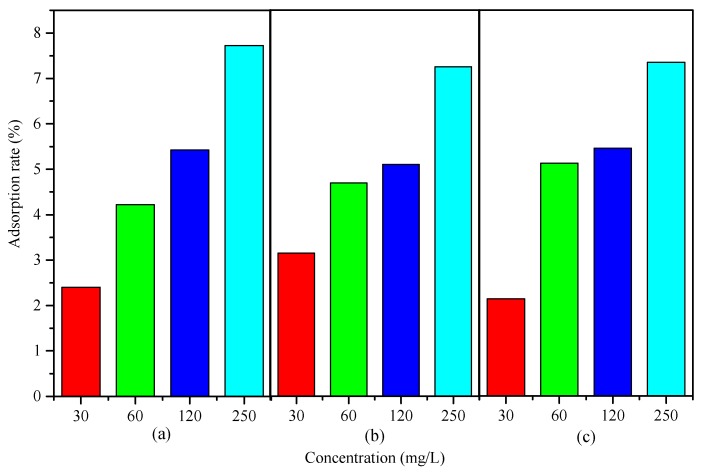
Effects of different anions on the iron adsorption rate. (**a**) The condition of sulfate; (**b**) The condition of chloride; (**c**) The condition of bicarbonate. Data were shown for duplicate experiments.

Therefore, the effects of sulfate, chloride, and bicarbonate on the iron adsorption rate after 12 h are shown in Figure 2.

The results indicate that iron could be adsorbed onto the inner wall of the PVC-U drinking pipe under the single factor condition of sulfate, chloride, and bicarbonate. It was also indicated in Figure 2 that the iron adsorption rate increased with the increasing concentration of sulfate, chloride. and bicarbonate. Moreover, the scales covering the inner wall of the PVC-U drinking pipe showed a rust-colored sight after adsorption. Compared to the stirring condition, iron was not as easily adsorbed on the inner wall of the pipes under a static condition. Under the static condition, iron was precipitated at the bottom of the pipe and could be washed off easily.

The reason for the adsorption could be explained by iron deposition [28]. For new PVC-U pipes, the three kinds of anions did increase the adsorption of iron. Within a short time, ferrous ion could be oxidized to ferric ion. The presence of the three anions significantly influenced the solubility of iron ion, thus improving the iron adsorption by PVC-U pipes.

### 3.3. Iron Release under the Single Factor of the Three Anions

According to previous studies, it has been demonstrated that goethite (α-FeOOH) and magnetite (Fe_3_O_4_) were the main components of the scales which covered the inner wall of old PVC-U drinking pipes [29]. The components of the scales on PVC-U pipes were similar to the dense layer of the corroded iron pipes [30,31]. These formed scales on the inner wall of the PVC-U drinking pipe which could be released into the water under certain conditions. The process of iron release from the prepared PVC-U drinking pipe mainly occurred in the initial 12 h, which was the same result obtained for the iron adsorption. Therefore, the trends of iron release from the inner wall of the prepared PVC-U drinking pipes under different water conditions after a 12 h reaction time was investigated in this part.

**Figure 3:**
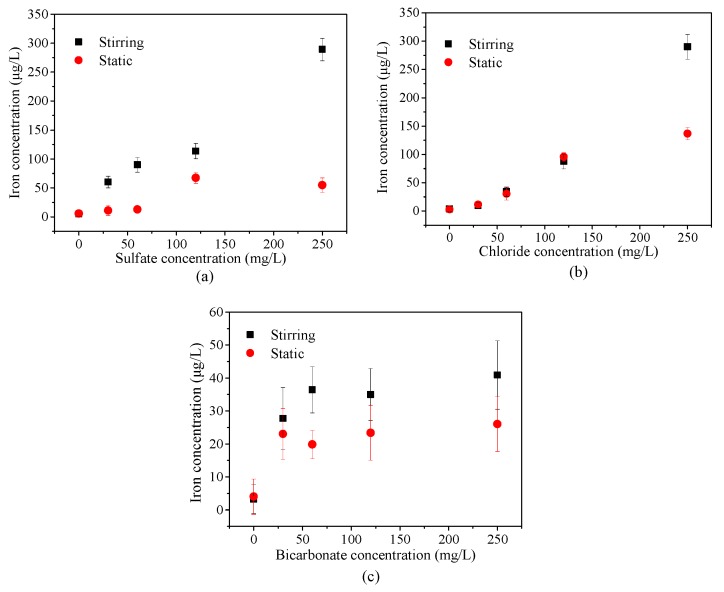
Effects of different anions on iron release. (**a**) The condition of sulfate; (**b**) The condition of chloride; (**c**) The condition of bicarbonate.

A higher amount of iron was released from the prepared PVC-U drinking pipes under the stirring condition when compared with the static condition, according to Figure 3. The reason for this is given by two interpretations. On the one hand, the stirring condition provided more oxygen to aggravate the reaction than the static condition, and therefore, the weight of the scales decreased with the increasing water velocity [2]; on the other hand, flow could hasten the precipitation of the protective layer and format to a denser protective layer, resulting in a decreasing iron release [1,29,32]. However, the protective scale could also be scoured away under an exceedingly high velocity.

In addition, the effects of sulfate, chloride, and bicarbonate on iron release from the prepared PVC-U pipe were different. Sulfate and chloride showed a similarly regular release with the increase of their concentrations. However, a higher concentration of bicarbonate had little effect on the release of iron. The presence of sulfate and chloride have been reported to promote the formation of lepidocrocite and goethite, which significantly affected the iron stability. Additionally, bicarbonate as an anion with a buffering capacity could form a dense and stable protective layer on the inner wall of the pipe, thus enhancing the iron stability [33,34,35].

### 3.4. Iron Release under Variability of LI

The LI index is the most widely used index in DWDSs for considering the anion effects on metal stability. Meanwhile, the LI index, as a synthetic indicator, showed that the ratio of chloride and sulfate to bicarbonate was important. The single effect of anions on iron release has been discussed in Section 3.3. However, the anions were present simultaneously under a natural condition. Previous studies have indicated that the higher the value of LI, the higher the corrosiveness of water [36]. After a chemical analysis of the experiment water, the tendency release iron was analyzed by the LI index. The experiments were designed using by RSM method. The details of the water samples (the experimental design matrix with anion variables) are presented in Table 1. The data were based on the 12 h reaction.

**Figure 4:**
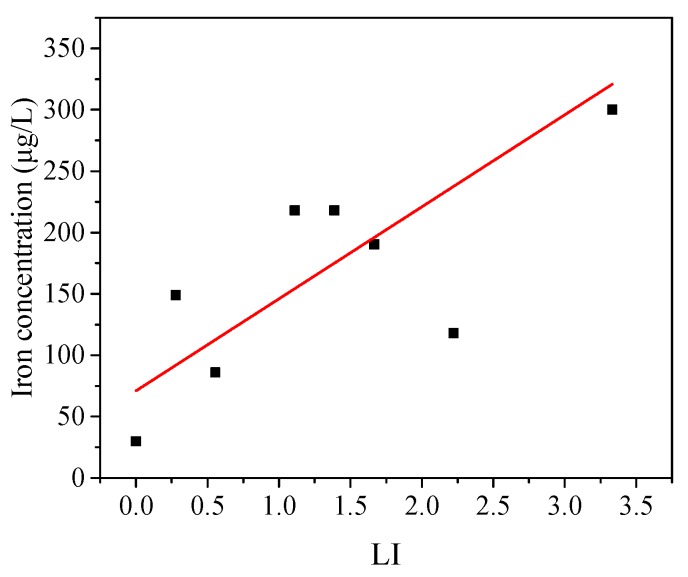
Iron concentration in water under different LI values.

Figure 4 indicates that the amount of iron release was positively correlated with the LI index. It could be concluded that increasing the value of LI might exacerbate iron release from the inner wall of the PVC-U drinking pipe. The simulated results demonstrated that the present regression model was a linear model, and the correlation coefficient (R^2^) was 0.544. In addition, the equation of LI (LI = ([Cl^−^] + 2[SO_4_^2−^])/[HCO_3_^−^]) indicated that the contribution of different types of anions (sulfate, chloride, and bicarbonate) to iron release was different. Furthermore, the contribution of sulfate to iron release was greater than chloride and bicarbonate, with a larger coefficient (2 to 1).

The LI index could be used to determine the corrosiveness of the water quality. However, the low correlation coefficient of the model meant that the accuracy of the model was not very good. Therefore, in order to better study and analyze the synthetic index of anions on iron release, it was necessary to establish a model to assess the iron release qualitatively and quantitatively.

### 3.5. Regression Models for Iron Release 

The effects of a single anion (sulfate, chloride, and bicarbonate) and synthetic indictor (LI index) on iron release were discussed above (Section 3.3 and Section 3.4). Compared with the LI index, the effects of different anions (sulfate, chloride, and bicarbonate) on iron release under a multi-factor water condition were investigated synthetically in order to produce a better regression model. Based on the experimental methods and data, three simulation models of iron release were compared, including a linear model, 2 factor impact model (2FI), and quadratic model. The details of the models are shown in Table 2 based on the 17 trials in Table 1.

Table 2 indicates that the theoretical model displaying the best fit for iron release was the linear model, with the largest value for the adjusted R^2^ and the lowest p among the three fitting models. Therefore, the linear model indicated that the effects of the interaction between the three types of anions on iron release were negligible compared with the single effect. The linear model in Table 2 was more efficient than the LI index (R^2^ was 0.724, compared to 0.544 for LI). The established linear model is shown in Equation (3).

(3) $$Y=81.6+0.56\times X_{1}+0.34\times X_{2}-0.069\times X_{3}$$

where *Y* (μg/L) is the concentration of iron in the test water after the reaction for 12 h; and *X*_1_ (mg/L), *X*_2_ (mg/L), and *X*_3_ (mg/L) are the concentration of SO_4_^2−^, Cl^−^, and HCO_3_^−^, respectively.

**Figure 5:**
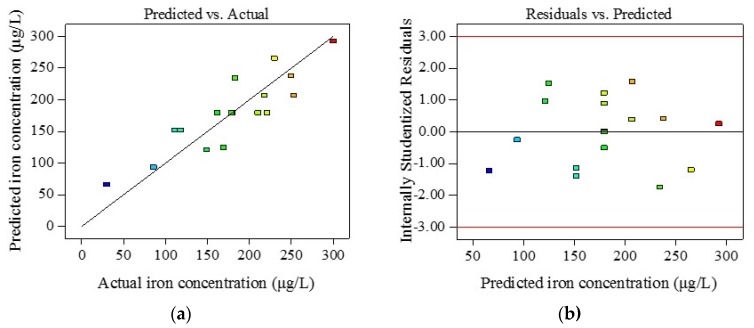
The relationship between the actual iron release and the model predicted one. (**a**) The actual iron release and model predicted values of the response variable; (**b**) The internally studentized residuals versus predicted values.

The summary of ANOVA in Equation (3) indicated that iron release under different water conditions could be well modified by a linear regression model. The correlation coefficient (R^2^) was 0.724 and the coefficient of variation (CV) was 19.72%, which are shown in Figure 5. Internally studentized residuals were used to recognize abnormal values. Figure 5b shows that all of the values were within the range of −1.743 and 1.57 (values beyond −3 and +3 were considered as the top and bottom outlier detection limits). The linear model established in this section confirmed a strong effect between iron release and the concentration of sulfate, chloride, and bicarbonate (Table 3). Therefore, the model could be used to predict iron release under the combined anions conditions, and was more accurate than the LI index.

From Equation (3), it could be concluded that the trend of iron release was positively correlated with the concentration of sulfate and chloride, but negatively correlated with bicarbonate. The results above correspond to previous researches which mainly focused on metal pipes [37]. In addition, Equation (3) also indicated that the contribution of sulfate to iron release was greater than chloride (for the bigger coefficient), which was consistent with the LI index. Thus, the results in Equation (3) also demonstrate that the linear model could be used to assess the iron stability on PVC-U drinking pipes. Additionally, the linear model exhibited a better fit than the LI index. Therefore, the method could calculate the trend of iron release from the inner wall of the PVC-U drinking pipe qualitatively and quantitatively, especially in the case of knowing the iron adsorption capacity on the inner pipe wall.

## 4. Conclusions

With the extensive use of plastic pipes, the process of iron adsorption and release on the inner wall of plastic pipes would significantly affect the iron content in water, which might affect people’s health.

In order to better analyze the effects of anions on iron stability in PVC-U drinking pipes, the effects of sulfate, chloride, and bicarbonate on the iron adsorption and release were studied. It was found that the iron adsorption rates increased with the increasing concentration of the three anions and could reach a balance within 12 h. For the simulated long-used PVC-U pipes, a different release profile appeared. Compared with the traditional LI index (a synthesized indicator for water chemistry), a well fitted linear model was established between iron release and the concentrations of sulfate, chloride, and bicarbonate. The established method played an important role in predicating the iron stability in PVC-U drinking pipes, which could be used in managing the drinking water safety for customers.

## Figures and Tables

**Table 1:** Experimental design matrix with anion variables.

Run Order		Variables (Water Chemistry)		
	SO_4_^2−^ (mg/L)	Cl^−^ (mg/L)	HCO_3_^−^ (mg/L)	LI
1	250	125	0	/
2	125	125	225	1.67
3	0	250	225	1.11
4	125	125	225	1.67
5	125	250	450	1.11
6	125	125	225	1.67
7	125	250	0	/
8	0	0	225	0
9	125	0	0	/
10	125	0	450	0.56
11	250	0	225	2.22
12	125	125	225	1.67
13	125	125	225	1.67
14	250	125	450	1.38
15	250	250	225	3.33
16	0	125	450	0.27
17	0	125	0	/

**Table 2:** Summary statistics of the models.

Source	*p*-Value	F Value	Adjusted R-Squared
Linear	0.0002	15.00	0.7241
2FI	0.7167	0.46	0.6848
Quadratic	0.8681	0.24	0.5911

**Table 3:** Analysis of variance (ANOVA) for the prediction of iron release.

Source	Sum of Squares	Mean Square	F Value	*p*-Value	Remark
Model	56,249.25	18,749.75	15.00	0.0002	significant
X_1_	14,706.13	14,706.13	11.76	0.0045	significant
X_2_	39,621.13	39,621.13	31.69	<0.0001	significant
X_3_	1922.00	1922.00	1.54	0.2370	not significant
Residual	16,254.63	1250.36			
Lack of Fit	13,889.43	1543.27	2.61	0.1845	not significant
Pure Error	2365.20	591.30			
